# Liver Biopsy in Metabolic-Associated Steatotic Liver Disease: Accuracy, Challenges, and Alternatives

**DOI:** 10.7759/cureus.103284

**Published:** 2026-02-09

**Authors:** Luca Borz-Baba, Adem Aydin, Russell Parvin

**Affiliations:** 1 Neuroscience, College of the Holy Cross, Worcester, USA; 2 Internal Medicine, Trinity Health of New England, Waterbury, USA; 3 Gastroenterology and Hepatology, Trinity Health of New England, Waterbury, USA

**Keywords:** fibrosis, liver biopsy, liver cirrhosis, liver elastography, magnetic resonance elastography (mre), metabolic-associated steatotic liver disease (masld)

## Abstract

Metabolic-associated steatotic liver disease (MASLD) is a common medical condition that can progress to metabolic-associated steatohepatitis (MASH) and advanced fibrosis, leading to severe outcomes. Liver biopsy continues to be the diagnostic gold standard; however, its accuracy and clinical utility in MASLD are subject to debate. Biopsy is also invasive and carries procedural risks. This contemporary narrative review examines the limitations of liver biopsy in MASLD and explores noninvasive alternatives. Our findings indicate that the diagnostic reliability of liver biopsy is limited by sampling error and by variability in tissue adequacy. Interobserver variability further adds to challenges in histological interpretation. Demographic factors and comorbid conditions significantly contribute to the misclassification of disease severity. Among well-established noninvasive tests, transient elastography and magnetic resonance elastography (MRE) assess larger liver volume and demonstrate dependable accuracy in detecting fibrosis, though both have implicit limitations. In conclusion, liver biopsy remains paramount in select clinical cases. Although current guidelines recommend noninvasive tests as first-line assessment tools in MASLD and MASH, no single approach is suitable for all patients. Future evaluation strategies should prioritize patient-centered assessments accounting for the multifactorial nature of MASLD.

## Introduction and background

Metabolic-associated steatotic liver disease (MASLD), previously termed nonalcoholic fatty liver disease (NAFLD), affects nearly 38% of the US population [1]. Its progressive form, metabolic-associated steatohepatitis (MASH), previously called nonalcoholic steatohepatitis (NASH), is characterized by inflammatory changes and fibrosis, with fibrosis recognized as the strongest predictor of mortality and disease advancement [2]. Although liver biopsy is considered the gold standard for grading inflammation and staging fibrosis, its accuracy is limited by multiple factors, including sampling variability, specimen inadequacy, histological interpretation, and interobserver differences among pathologists [3]. This narrative literature review aims to synthesize existing contemporary evidence on the limitations of biopsy in MASLD, including technical and interpretive challenges, patient-level implications such as complications and psychological burden, and the risk and clinical impact of misclassification. The review concludes by describing emerging noninvasive diagnostic alternatives and outlining the clinical context in which liver biopsy remains warranted in MASLD.

Literature search and review scope

Based on the objectives described above, we conducted a directed literature search in PubMed/Medical Literature Analysis and Retrieval System Online (MEDLINE), Embase, and Scopus, including publications from January 2005 to December 2025. We selected relevant studies and major society guidelines. By design, this narrative review does not follow a systematic review methodology or meta-analysis format.

## Review

Accuracy of liver biopsy in MASLD

Sampling Error and Specimen Quality

Sampling error contributes to significant discrepancies in liver biopsy, as only 0.002% of the liver is evaluated in one biopsy sample. The length of the biopsy specimen is critical, as samples larger than 25 mm (that include 6-10 portal tracts) are associated with the highest detection rate of MASH (65%), whereas smaller samples (<10 mm) detect MASH in only 29% of cases, emphasizing that diagnostic accuracy is highly dependent on the size of biopsy sample [4]. The diameter of the specimen additionally plays an important role. Since the average liver lobule measures 0.5-1 mm, specimens with an inner diameter of <1.2 mm are considered suboptimal and can lead to lower levels of accuracy in fibrosis staging [4-6].

When feasible, obtaining multiple cores increases accuracy. The standardization of tissue processing is critical, yet these protocols may be inconsistently applied, especially in community-based hospitals where training or resources may fall short to that of academic centers [7]. In a study conducted by Fryer et al., specimen inadequacy was reported as high as in 23.8% of cases [8]. Interpretive variability adds another dimension to diagnostic inaccuracy. Community-based pathologists have been shown to understage fibrosis compared to specialists in tertiary centers [9]. These findings reveal that many centers may not meet standards and suggest the need for a universal regulatory requirement for standardized processing.

Histological Interpretation

Although fibrosis is the strongest predictor of outcomes in MASLD, the diagnostic value of liver biopsy also relies on the identification of steatosis, lobular inflammation, and ballooning degeneration. These features form the basis for scoring systems used for diagnosis and disease management [3,10]. Over the years, several composite scoring systems have been developed: NAFLD Activity Score (NAS), which is endorsed by the American Association for the Study of Liver Diseases (AASLD) [11], and the Steatosis, Activity, and Fibrosis (SAF) score [12-14]. The SAF score, most commonly utilized in Europe, was designed to provide a structured diagnostic and grading framework of MASH.

Despite the development of numerous scoring systems, a multicenter Korean study published in 2016 reported that no single histopathological system is universally reliable [15]. The subjective nature of histological interpretation can lead to variability in the recognition and grading of key lesions [16]. Ballooning and inflammation are particularly noted to demonstrate high variability, while steatosis is subject to the least variability, and fibrosis has the highest interobserver concordance [12,15,17]. To mitigate the limitations of subjective scoring systems, algorithmic approaches such as the Steatosis, Activity, and Fibrosis/Fatty Liver Inhibition of Progression (SAF-FLIP) have been proposed; however, none have been formally incorporated in AASLD guidelines to date [3,16].

The uneven distribution of steatosis, inflammation, and fibrosis across the liver furthermore challenges accurate diagnosis and staging [6,18]. To address this, larger or multiple biopsy samples can be obtained to improve diagnostic accuracy [19]. However, this strategy is not routinely adopted [6]. Integrated machine learning models have been proposed as a tool to help reduce interobserver variability in MASLD.

Histological interpretation can also be challenging due to overlapping histological features across liver diseases. For example, portal inflammation is seen in MASLD but can mimic autoimmune hepatitis or autoimmune-MASLD overlap [20,21]. Similarly, despite differing by etiology, fibrosis exists in all stages of liver disease. However, fibrosis in alcohol-related liver disease demonstrates perivenular and centrilobular distribution, whereas MASLD is characterized by perisinusoidal fibrosis and is most pronounced in zone 3 [22]. Nevertheless, these features can coexist in the same patient, further blending boundaries. Therefore, guidelines emphasize that biopsy findings must be interpreted within a clinical and biochemical context [3,10].

These findings demonstrate that despite standardized scoring systems and algorithms, histological interpretation in MASLD remains complex and not consistently reliable.

Integrated machine learning models have been proposed as a tool to help reduce interobserver variability in MASLD, specifically by using the second harmonic generation/two-photon excitation (SHG/TPE) technique. In a research setting, SHG/TPE microscopy is able to provide detailed images and quantification of fibrosis on unstained specimens. This novel digital pathology tool merits further investigation, as eliminating variation in slide preparation could enhance the accuracy of fibrosis assessment [23].

Pathologist Expertise

Pathologists with specialized training in liver diseases are more likely to provide accurate diagnoses compared to general pathologists [13]. European practice guidelines recommend that liver biopsies be preferably interpreted by or referred to hepatopathologists with expertise to decrease interobserver variability [19,24]. AASLD supports this recommendation but notes that such expertise is often unavailable in practice. In addition, in a national survey among newly hired pathologists, only 9% of those in community practice reported subspecialized training, underscoring the large gap in expertise available between university or tertiary centers and community practice [25]. This highlights the need for consistent collaboration to increase reliability and strengthen referral pathways.

If SHG/TPE changes the biopsy substrate, the 2025 consensus recommendations from the International MASLD Pathology Group emphasize changing the process or workflow. The experts proposed centralized reading by two independent pathologists, with a third reader being required to adjudicate in case of pathologist disagreement. They also endorse standardized training modules for the pathologists and periodic calibration sessions. The consensus promotes the use of digital images and telepathology, allowing remote review and sharing of imaging between centers to increase inter-rater agreement [26].

Impact of Patient Demographics and Comorbidities on Histological Findings

Research has shown that elderly patients are more likely to demonstrate moderate to severe fibrosis, men and postmenopausal women with MASLD have a greater risk of developing fibrosis, and Hispanics have a higher risk for MASH compared to Caucasians or Black despite similar fibrosis severity [27-31]. These demographic characteristics function as risk modifiers that influence disease prevalence and pretest probability, reiterating the importance of interpreting liver biopsy findings within the appropriate demographic context.

Patient-related comorbidities, such as obesity, diabetes, and metabolic syndrome, can further complicate the interpretation [32]. Obesity increases intrahepatic heterogeneity, as demonstrated in bariatric surgery cohorts, increasing the risk of under-staging fibrosis [33]. Comorbidities such as diabetes, chronic kidney disease (CKD), and congestive heart failure (CHF) can also exaggerate steatosis, inflammation, and fibrosis, consequently reducing interpretive reliability [34-37].

Although comorbidities are incorporated in clinical scoring systems such as Fibrosis-4 (FIB-4) Index and NAFLD and contribute to referrals for more advanced testing, such as elastography or biopsy, histological scoring systems, although standardized, do not account for comorbidities. These conditions also act synergistically to accelerate MASLD progression [36]. Finally, the severity captured on a single biopsy may not accurately represent the true state of the disease. Prospectively, a comprehensive approach to patient evaluation that considers comorbid conditions is essential for the accurate assessment of the severity of liver disease.

Clinical and patient implications of biopsy

Risks and Complications

Despite being a key diagnostic resource, liver biopsy still carries risks, including pain, bleeding, cholangitis, and, in rare cases, hospitalizations (0.65%) or mortality (0.01%) [38]. Major complications arise in approximately 2.4% of cases according to a meta-analysis conducted by Thomaides-Brears et al. [38].

While the overall risk of major bleeding after percutaneous biopsy is low (~0.48%), bleeding deserves particular attention as a risk, as its associations with age differ by series [38]. A large meta-analysis found that patients of younger age are associated with higher hospitalization due to major bleeding. Consequently, age-related effects should also be considered with caution, while modifiable risk factors, such as passes and coagulation status, should be optimized.

Coagulopathy, reflected by an elevated international normalized ratio (INR) and thrombocytopenia have long been debated as risk modifiers. In a 2010 report of over 2,500 patients with hepatitis C who had liver biopsy, bleeding risk was higher in patients with a platelet (PLT) count of ≤60,000/µL or an INR of >1.3 [39]. However, the current AASLD guidelines state that INR or platelet count alone should not determine bleeding risk [40]. British societies use a cutoff for INR correction of >1.4 and platelet count of 50×10⁹/L [41]. According to AASLD, when the bleeding risk for percutaneous liver biopsy is high, transjugular liver biopsy is preferred [42]. Also, patients with advanced CKD are particularly vulnerable due to platelet dysfunction. These considerations highlight the need for individualized risk assessments.

Psychological Distress in Patients

Patients with MASLD and MASH have higher rates of depression and generalized anxiety disorder and therefore may experience psychological distress undergoing liver biopsy [43]. Psychological distress is specifically driven by procedural fears, uncertainty around potential complications, and the implications of results [44]. Addressing these psychological concerns through clear communication regarding the risks and benefits of the procedure can improve the patient experience. Additionally, structured pre-procedural counseling and support may help alleviate anxiety and streamline patient care.

Consequences of Misclassification

Misclassification can occur through real histological mis-staging (secondary to heterogeneous disease distribution and sampling error) or in cases of technically accurate biopsies, through clinical misinterpretation when demographic characteristics or comorbidity-related risk modifiers are not considered, leading to inappropriate over- or under-staging. Under-staging may delay essential interventions, while over-staging can expose patients to unnecessary treatments or monitoring [45]. As of 2024, patients with MASH and moderate to advanced fibrosis (F2-F3 fibrosis) are eligible for treatment with resmetirom. Therefore, accurate staging has transitioned from the academic realm and research setting to the real-world clinical practice, where it is critical for obtaining insurance approval and in avoiding treatment delays [46].

Alternative diagnostic methods

Imaging Tests

In clinical practice, serum-based tests, specifically Fibrosis-4 (FIB-4) Index, are used as an initial step to stratify fibrosis risk and guide referral for further evaluation with biopsy or alternative diagnostic methods. Given the challenges associated with liver biopsy, imaging tests such as Vibration Controlled Transient Elastography (VCTE) and magnetic resonance elastography (MRE), which assess liver stiffness to give insight into fibrosis severity, have been evaluated as alternative diagnostic tools [47]. Unlike biopsy, which samples a small portion of the liver, these methods analyze the entire organ, thereby counterbalancing sampling error. VCTE demonstrates high accuracy for identifying advanced fibrosis (fibrosis stages F3-F4) but lower accuracy in detecting F0-F2 fibrosis [48,49] and is influenced by factors such as obesity, ascites, fluid overload, and coexisting hepatitis C infection [50]. Compared to VCTE, MRE demonstrated superior sensitivity and specificity for all stages of fibrosis (F0-F4), quantifying steatosis, and can distinguish between them [50,51]. Clinical guidance recommends VCTE as the first-line test, reserving the use of MRE for cases in which VCTE results are inconclusive [3]. Given the well-established noninvasive prognostic tools and the limitations of histological interpretation, liver biopsy is reserved for selected cases, specifically when the diagnosis remains uncertain, noninvasive studies generate conflicting results, or they suggest aggressive liver disease in the absence of typical metabolic risk factors [3].

Clinical Scenarios in Which Liver Biopsy Remains Indicated

According to AASLD 2023 guidelines, liver biopsy is in the differential diagnosis of steatosis, including steatosis from Wilson disease for quantitative copper measurement, when noninvasive assessment indicates fibrosis of ≥F2 in patients with high risk for progression to cirrhosis, noninvasive evaluations lead to inconclusive results or are discordant with the clinical presentation, aminotransferases are persistently elevated (>6 months) or additional or alternate diagnosis is suspected (iron overload and drug-induced liver injury), etc. [3].

## Conclusions

The diagnostic value of liver biopsy in patients with MASLD and MASH is influenced by a multitude of factors, including sampling error, specimen adequacy, histological interpretation, pathologist experience, and comorbidities. Clinical and patient implications, such as complications, psychological impact, and the consequences of misclassification, should all be considered prior to biopsy. While noninvasive investigations remain the first-tier approach in MASLD, biopsy continues to play a selective role in diagnostic dilemmas or when results are discordant. Accurate fibrosis staging is now even more critical for timely access to therapy, such as resmetirom, which was approved for patients with MASH and moderate to advanced fibrosis. In this context, digital pathology and AI-assisted avenues promise to improve the reproducibility and standardization of histological interpretation and help preserve the clinical value of liver biopsy within an evolving MASLD landscape.
